# Are patient-centered care values as reflected in teaching scenarios really being taught when implemented by teaching faculty? A discourse analysis on an Indonesian medical school's curriculum

**DOI:** 10.1186/1447-056X-10-4

**Published:** 2011-04-25

**Authors:** Mora Claramita, Adi H Sutomo, Mark A Graber, Albert JJ Scherpbier

**Affiliations:** 1 Department of Medical Education, Faculty of Medicine, Gadjah Mada University, Jalan Farmako, Sekip Utara No 1, Grha Wiyata Building 3rd floor, Yogyakarta - Indonesia; 2 Faculty of Medicine, Gadjah Mada University, Jalan Farmako, Sekip Utara No 1, Radiopoetro Building 1st floor, Yogyakarta - Indonesia; 3 Department of Family and Emergency Medicine, University of Iowa, Carver College of Medicine, USA; 4 Institute for Education, Faculty of Health and Life Sciences, Maastricht University, The Netherlands

**Keywords:** Patient-centered care, Comprehensive care, Continuous care, Discourse analysis

## Abstract

**Background:**

According to The Indonesian Medical Council, 2006, Indonesian competence-based medical curriculum should be oriented towards family medicine. We aimed to find out if the educational goal of patient-centered care within family medicine (comprehensive care and continuous care) were adequately transferred from the expected curriculum to implemented curriculum and teaching process.

**Methods:**

Discourse analysis was done by 3 general practitioners of scenarios and learning objectives of an Indonesian undergraduate medical curriculum. The coders categorized those sentences into two groups: met or unmet the educational goal of patient-centered care.

**Results:**

Text analysis showed gaps in patient-centered care training between the scenarios and the learning objectives which were developed by both curriculum committee and the block planning groups and the way in which the material was taught. Most sentences in the scenarios were more relevant to patient-centered care while most sentences in the learning objectives were more inclined towards disease-perspectives.

**Conclusions:**

There is currently a discrepancy between expected patient-centered care values in the scenario and instructional materials that are being used.

## Background

The "Health for All" goals as stated in the 1978 Alma Ata declaration described the need to acknowledge the patient as a person [1]. It is essential that patient-centered care be recognized by health professionals who work in any domain of medicine. Patient-centered care is at the core of care provided by family practitioners [2-7]. Caring for patients, while recognizing their personal and environmental background helps to define the specialty of family practice. This is in contrast to other specialties that often view patients based on particular specific diseases.

In 2006, the Indonesian Medical Council, adopted a competence-based education as the basis for undergraduate medical curriculum and clearly stated the orientation of the curriculum should be towards family medicine [8-10]. The idea was that all undergraduates would learn 7 competencies: effective communication, clinical skills, medical knowledge, patient-management, information-management, life-long learning and ethics-professionalism [8]. Looking at general scope of the 7 competences, patient-centered care values should have already been included.

There is often a discrepancy between "curriculum on paper" and "curriculum in action" [11]. One study from the Netherlands found that medical students in their last year of school viewed disease oriented learning more favorably than behavioral and social sciences [12]. One study from Australia found that there was a conflict between desired learning outcomes towards elderly health problems; instead of training in chronic diseases, the actual scenarios of tutorials mostly leaned towards more acute illnesses [13]. A lesson learned from a European medical school, which used a problem based learning curriculum since its establishment in 1974, showed that a consistent and persistent focus of the final objectives of the curriculum towards family medicine was important but easier said than done; there is a creep towards disease oriented training [14]. Other studies using exploratory approaches uncover the hidden agenda within a curriculum which were more teacher-centered, examination driven, and required passive learning on the part of the student [15,16]. A more disease oriented training persists within the medical curriculums in both Western and non-Western medical schools.

Inspired by those lessons, we have chosen to find out if the scenarios and learning objectives in an Indonesian medical school's curriculum that oriented towards family medicine practice were adequately sensitizing students to the values of patient-centered care. We used a qualitative approach by applying a discourse analysis method to explore any discrepancy between problem formulation in the scenarios (presented to the students) and its learning objectives (hidden and driven by tutors) towards a patient-centered care tutorial discussion.

## The context of this study

### The New Issue of Family Medicine in Indonesia

In most of the world, education in family practice is taken at the post graduate level [17]. Family practice specialization in the Asian region takes 1 to 3 years of formal, structured postgraduate education program for general practice or family practice specialization. The exceptions are Indonesia and few other countries [2].

The current national health care and health education system in Indonesia requires six years of undergraduate medical education before becoming a physician. Planning included an additional year of internship. However, clinical education in Indonesia was department-based and there are no recognized departments of family medicine or general practice in any of the medical schools in Indonesia. It is a classic "chicken and egg dilemma": we want training programs in family medicine but there are no specialists in family medicine to teach our undergraduates and graduates. Thus, teachers for the internship program may come from general practitioners who do not possess adequate formal postgraduate education on family medicine or may come from hospital-based specialties that do not reflect the realities of health problems in the community. There are gaps in time-spent, knowledge, skillfulness and professionalism between GPs and other specialists in Indonesia.

Approximately two thirds of the doctors in Indonesia are primary care doctors. Considering the essential roles of primary care doctors in this country and the lack of postgraduate clinical training, the World Health Organization (WHO) made a recommendation in 2003 that Indonesian primary care doctors should undertake additional postgraduate education as family doctors [2]. However, the representative board of Indonesian Medical Council decided not to go along with this recommendation. Rather, they decided to improve the training in "Family Medicine" at the undergraduate level.

### The development of a competence-based curriculum that oriented towards family medicine using a problem based learning approach in an Indonesian school of medicine

In this study, we examined an Indonesian competence-based medical curriculum. The curriculum oriented towards family medicine practice and uses a problem based learning approach to stimulate self directed learning in which adapted from a Western medical curriculum [14]. We chose to examine a curriculum of a medical school which has the longest tradition of medical education innovation in this country. This medical school has been using a problem based learning strategy since early eighties and has led the curriculum development of many other health education institutions in Indonesia. In this medical school, the 7 areas of competences are in theory adapted into many learning strategy such as tutorial discussions, skills training, laboratory practice and field work [18].

A macro view of the curriculum shows a design around general learning objectives (based on the 7 competencies from Indonesian Medical Council, 2006) for each academic year. The themes for year 1, 2 and 3 are: The Human Body System, The Life Cycle and The Multisystem or Chronic Illnesses consecutively. Each academic year theme consists of 6 blocks. Afterwards, an assessment committee guided the general guidelines of the block assessment process.

At the micro level of the curriculum, the multidisciplinary block planning group designed each block, starting from re-considering the learning objectives, creating the scenarios for discussion, defining the facilitators (tutors, lectures, and instructors), organizing the schedule and finally creating scenarios for the assessment as guided by the assessment committee. Each blocks containing 6 scenarios. One scenario may consist of 5-10 learning objectives known to the tutors but not to the students.

## Methods

We analyzed the discourse in the scenarios and the learning objectives of year 1, 2 and 3. We did not include year 4, year 5 and year 6 because they were under construction during the study period. A discourse analysis is chosen to provide rich analysis on the event and meaning beyond the analyzed-texts [19-21].

### Subjects

Three coders were general practitioners from a family medicine team at an Indonesian school of medicine. The coders work in a routine primary care service and are involved in the teaching of primary care for undergraduate medical students for more than 10 years. As one of coders, the first author underwent a distance socio-cultural learning theories course (included discourse analysis course) for 9 months provided by the School of Health Professional Education, Maastricht University, The Netherlands, 2010-2011. Other coders were trained in discourse-analysis methods by the first author for a total of 16 hours using international guidelines [19-21]. We analyzed scenarios and its learning objectives of a total 108 scenarios and 1080 learning objectives of the 18 blocks in year 1, 2 and 3.

### Instruments

We used two principles representing the patient-centered care which are fundamental for primary care practices: 1. The Comprehensive Care based on the Bio-psycho-social-economic-education-environment-culture and spiritual concern towards patients and 2. The Continuous Care based on adequate understanding of five levels of prevention and the natural history of diseases [4-7]. We defined two categories in the coding process: met or unmet the educational goal of patient-centered care. We defined it "met educational goal of patient-centered care" if the sentence stimulated students to think beyond the disease, more about the patient with his/her illness and perspective, the environment and also continuity of care. The three coders met three times 2 hours to agree on certain criteria considering comprehensive care and continuous care, before starting the coding process. The agreement on general criteria was presented in Table 1.

**Table 1:** Agreement among three coders of met and unmet educational goals of patient-centered care criteria

"Comprehensive Care" principles	"Continuous Care" principles
The sentence should stimulate students to think and discuss about the patient as a person: 1. Begin with patients' personal identity to deeply explore patients' family and family interaction - time, place, people. 2. Begin with patients' personal identity to deeply explore patients' daily activities and environmental circumstances 3. Begin with patients' personal identity to deeply explore patients' psychological background	The sentence should stimulate students to think and discuss about the importance of rich exploration of history of illness or pre hospital history (including family history and risk assessment)
The sentence should stimulate students to think and discuss about the need of exploration skills to be sensitive to patients 'concern (listening ability, empathy, interpersonal skills, open and close questions, paraphrasing, facilitating story).	The sentence should stimulate students to think and discuss 5 levels of prevention (including plans and regular assistance after hospital)
	The sentence should stimulate students to think and discuss health risk assessment across ages.
	The sentence should stimulate students to think and discuss about the need of negotiation skills with patients (e.g. to help making sense of their illness, to obtain consent, to discuss alternatives and difficulties, to do informed and shared decision making).

### Procedures

Each coder defined each sentence in each scenario and learning objectives of each block, based on the two categories. Three coders met when they finished 6 scenarios in 1 block to discuss their findings of the two categories until they reached agreement. Total coding process took 20 weeks (April-August 2010). The coding process took place in Bahasa Indonesia. The first author translated the result into English for presentation and manuscript writing purposes, after agreement of other coders.

### Analysis

The text in scenarios and learning objectives was analyzed based on discourse analysis methods [19-21].

## Results

Results are noted in Table 2. All three coders agreed that 527 sentences among 650 sentences in the 108 scenarios met the educational goal of patient-centered care. There were 1080 sentences in the actual teaching material. Of these, only 104 of the sentences met the educational goal of patient-centered care. Table 3 described examples of discourse-analysis findings of the scenarios and the learning objectives of an Indonesian medical school's curriculum. We found a discrepancy between written scenarios, which mostly addressed patient-centered care principles (the comprehensive and continuous cares) and the implemented learning objectives in which leaned towards specific diseases or medical content.

**Table 2:** Agreement among three coders of number of scenarios and learning objectives analyzed by discourse analysis method

Sentences analyzed by Discourse methods	Academic Year	Educational goal of patient-centered care: Comprehensive and Continuous Care		
		Met	Unmet	Total sentences
In the Scenarios	1	56	58	
	2	207	37	
	3	264	28	
Total		527	123	650
In the Learning Objectives	1	31	389	
	2	46	328	
	3	27	259	
Total		104	976	1080

**Table 3:** Description of discourse-analysis findings of three scenarios and its learning objectives from three academic years of an Indonesian medical school's curriculum

Sentences in scenarios of a block in each academic year	Learning objectives of particular scenario stated in Tutor's Book	Agreement among three coders about the discrepancy of patient-centered values between the scenarios and the learning objectives
**Taken from a scenario in year 1- Block 1.3 Digestive system** **Year theme: Human Body System** **Title: Sunken Eyes** **Sentence 1: **A male baby, 11 month was brought to primary health care with diarrhea and vomiting for two days. **Sentence 2: **His mother usually giver him formula milk in a bottle. **Sentence 3: **He was irritable, eager to drink and had sunken eyes. **Sentence 4: **The midwives in the PHC assessed him to some degree of dehydration and gave him oral rehydration solution. **Sentence 5: **Please think a lot about the mechanism of the problem above.	1. Anatomy and physiology or small and large group intestine 2. The role of digestive enzymes 3. Biochemical digestive and absorption process of dietary food 4. Microstructure of digestive enzymes producing cell 5. Physiology of digestion and absorption of carbohydrate, lipid, protein, minerals and water) 6. Biomedical importance of carbohydrate 7. Biomedical importance of protein 8. Biomedical importance of lipid 9. Pharmacokinetic and pharmaco-dynamic 10. Basic of virology 11. Normal fluid consumption 12. Influence of drug route administration and drug onset and duration	General agreement on Tuesday April 13^th ^2010: None of the learning objectives in this scenario addressing the patient's background, patient's problem or patient's family and environmental condition. It is clear that there is a gap of patient-centered care between scenario and learning objectives. The theme of year 1 should be expanded to more than "a human body system" and more to address the family as a unit of care. Agreement on the Comprehensive Care value: In sentence 2 in the scenario, there is a wide opportunity to explore the background of the patient and his mother. Possible questions were: Why the mothers prefer to give him formula milk? What was she doing every day, did she take-care of her baby by her selves or there is somebody else help her? Did she have to work full time a week? What about her husband, the grandpa and grandma and the supportive neighborhood that was characterized this society in raising a baby? However, no learning objective seemed to support this direction of patient-centered care. The coders understand that for the first year students, basic medical sciences such as anatomy, biochemistry and physiology were fundamentals. However, a curriculum that oriented towards family medicine should also address the family as a unit of care, since early basic medical curriculum. Agreement on the Continuous Care value: Sentence 3 and sentence 5 was rather contradictory. Sentence 3 may stimulate students to think of the natural history of such illness and may facilitate thinking of how to provide preventive care so that such a case would never happened again to the baby. However, before students could think a bit about preventive care and the natural history of disease of an acute illness such as diarrhea, sentence 5 directly drove students to learn basic sciences of anatomy, physiology and biochemistry, which is of course important for first year students, but may drive the tutorial discussion away from a patient-centered care.

**Taken from a scenario in year 2- Block 2.3 Infancy and childhood** **Year theme: Human Body System** **Title: Sunken eye girl** **Sentence 1: **A two years old female child was brought to an outpatient clinic because of diarrhea. **Sentence 2: **She had watery diarrhea for 15 days and passed stool about 7 times a day. **Sentence 3: **There was neither mucus nor blood in the stool. **Sentence 4: **Her mother had tried to give her oral rehydration solution. She was eager to drink but vomit afterwards. **Sentence 5: **On physical examination she looked restless and irritable. **Sentence 6: **Her eyes were sunken and abdominal skin pitch went back slowly. **Sentence 7: **She was very think and her body weight was 8 kilograms. **Sentence 8: **The doctor found diaper rash. **Sentence 9: **Her eyes were sunken and abdominal skin pitch went back slowly. **Sentence 10: **She was hospitalized in order to manage her dehydration. **Sentence 11: **A further assessment into child's case found that she had been hospitalized several times because of diarrhea and respiratory tract infection.	1. Explain the definition of diarrhea 2. Explain the etiology of diarrhea 3. Recall of water and mineral absorption in the gut 4. Explain the pathophysiology of viral and bacterial diarrhea 5. Explain the degree of dehydration 6. Explain the management of diarrhea based on guideline of WHO 7. Explain the complication of severe dehydration including electrolyte imbalance, acid-base imbalance and hypovolemic shock 8. Describe the cause of toxin induce food borne illness 9. Define lactose intolerance 10. Explain the composition of oral rehydration solution 11. Explain the management of persistent diarrhea 12. Explain hypernatremie 13. Explain about vomiting 14. Explain about constipation 15. Explain about abdominal distension 16. Explain jaundice	General agreement on Tuesday July 13^th ^2010: It is good that similar case was repeated again in the second year to show the spiral based education. This case may stimulate students' prior knowledge and adjust more knowledge in the same area. Agreement on the Comprehensive Care value: Sentence 2 should facilitate students to think more about the background of this child and her family. Based on the year theme Life Cycle, possible questions are: Into what stages this child had grown up physically and mentally based on natural child growth and development? Why after 15 days the child was brought to a clinic? Why not immediately after she found diarrhea? What is the underlying reason of her parents? Did they face difficulty on social economic or something else? Who take care of the baby every day? What is happening during the last 15 days? However, such curiosity on patients' bio-psycho-socio-eco-educ-cultural background did not supported by the learning objectives of this scenario. All 16 learning objectives may drive students to think about only medical knowledge rather than deepen their curiosity on patient-centered care issues. Agreement on the Continuous Care Care value: The last sentence in the scenario should facilitate students to think about underlying illness. A child who often hospitalized because of diarrhea and respiratory infection may have an underlying disease which is important to explore. The main possible question is: How a doctor that oriented towards family medicine helps to prevent the particular illness from falling into further stage in the natural history of particular illness? Moreover, a doctor that oriented towards family medicine should put attention on child's normal development stages and possible influence of this underlying disease. It is a misfortune that all 16 learning objectives did not support this preventive and promotive care in which vital for primary cares doctors.

**Taken from a scenario in year 3- Block 3.3 Abdominal complain** **Title: Bloody stool** **Sentence 1: **A 50 years old female came to her physician for a routine health maintenance examination. **Sentence 2: **On physical examination, there were no remarkable findings but a stool sample was positive for occult blood. **Sentence 3: **Doctor planned to do further laboratory examination for tumor marker's identification and colonoscopy. **Sentence 4: **The patient refused further treatment and workup. **Sentence 5: **Ten months later she visited her physician because of constipation, micrositic anemia and 5 kg lost of body weight in the last 3 months. **Sentence 6 **On physical examination, there were hepatomegaly slight ascites. Ultrasonography shows multiple mass in the liver.	1. Explain the epidemiology, the natural history, symptom and sign, the diagnosis and management of colorectal cancer 2. Explain the association of the cancer with fiber diet 3. Emphasize the importance of endoscopy in dyspepsia 4. Emphasize the importance of tumor marker examination in colorectal cancer 5. Explain the staging and grading of the colorectal cancer.	General agreement on Tuesday 24 August 2010: This is a "good" scenario to show the perplexity of guiding students into basic medical sciences or primary care or hospital based level. The case was often found in the hospital and it seemed intended to show how primary care level had failed. It is a good example when the teacher knows to facilitate students to prevent such a terminal illness. However, without careful attention, students may not realize that this is the primary care physician's task and responsibility and so he/she should have an excellent relationship with the patient to gain her trust and cooperation for further clinical decision making. Agreement on the Comprehensive Care value: Sentence 1 in the scenario shows that this patient was on routine general check up. It means that she was possibly aware of the importance of general check up and she has adequate health insurance to support, particularly in this context of this study where fee for services usually out of pocket. Sentence 4 shows that this patient did not want to follow the procedure for further examination. Here comes a big question: Did the patient was well-informed about the possibility of the result of a general check up and the consequences? A middle age women who undergone routine checkup - (every year?) - should be well informed about the consequences of a medical checkup. This is one of the main tasks of a doctor who oriented towards family medicine to shares information; before the illness become advances and before a further clinical decision was made. If situation in the sentence 4 happens, doctors should explore the reasons why this woman refuses for further examination. Good interaction skills between doctors and patients are necessary and should be stated as one of learning objectives. So, students could emphasize on illness perspective and not only focus on the disease perspective as stated on the learning objectives. Agreement on the Continuous Care Care value: If situation in the sentence 4 happens, doctors should shares information about the natural history of diseases of particular illnesses as a consequence of the result of the current general check up. The patient should be well-informed and a written informed consent maybe necessary. This process is an important task of a doctor to prevent the patient from falling into further stage of illnesses as expressed in sentences 5 and 6. This process of shares information and shares decision should also be stated as one of learning objectives. We identified that the task of a doctor who oriented towards family medicine, to provide/assist optimum (if it cannot be maximum) prevention to the patient was not adequately comprehended in this scenario and its learning objectives. Sentence 5 and sentence 6 in the scenario seemed to adequately addressing the issue of prevention, but most likely to be fail by the time students saw the learning objectives.

Examples of sentences in the scenarios that met the educational goal of patient-centered care values:

"*She plans to get married next month but she would not like to get pregnant until she finishes her study in the Faculty of Law*." (Sentence-3, Scenario-4, Block 2.2 Safe Motherhood and Neonates)

"*His doctor has an appropriate recommendation for his work and family counseling after recovery*". (Sentence-9, Scenario-3, Block 2.5 Adulthood)

"These sentences stimulate students to think beyond patient's health problems. The patient-centered care values were adequately expressed in the concern of comprehensive care and the continuous care towards the patient as individual, as family member and as a member of his/her society".

(Agreement among three coders)

Examples of sentences in the scenario that unmet the educational goal of patient-centered care values:

*"Please think a lot about the mechanism of the problem above"*. (Sentence-5, Scenario-2, Block 1.3 Digestive System)

*"The doctor sent her to the laboratory for a skin-scrapping and a direct skin examination test by KOH 30% stain and culture in order to find out the causative agent"*. (Sentence-6, Scenario-7, Block 2.5 Adulthood)

"These sentences may drive students to think on the pathophysiology and procedures, in which of course fundamental for basic medical sciences; however less attention may be given to the patient as a person. The first sentence was the last sentence in the particular scenario that confronted all patient-centered messages in previous sentences. The second sentence mentioned the word 'sent' without careful acknowledgement on patient's consent". Carefully obtaining patients' consent is fundamental in comprehensive care as well as continuous care principles. Obtaining patients' consent may become one of learning objectives in this scenario, so that students may not only pay attention on the disease (diagnosis and treatment) but also paying attention on patient's concern.

(Agreement among three coders)

Examples of sentences in the learning objectives that met the educational goal of patient-centered care values:

"Aware of the professional value as a medical doctor."

(Learning Objective-5, Block 1.1 Locomotors System)

"Conduct physical examination in a holistic manner according to patient's problem".

(Learning Objective-3, Block 3.4 Limited Movement)

"These sentences stimulate students to think beyond patient's health problems. The patient-centered care values were adequately expressed the personal care in the behavioral-socioemotional ethical concern towards patient as a person".

(Agreement among three coders)

Examples of sentences in the learning objectives that unmet the educational goal of patient-centered care values:

"Explain etiology and classification of heart failure-New York Heart Association".

(Learning Objective-3, Block 3.2 Chest Complaints 2009)

"Explain the definition of diarrhea".

(Learning Objective-1, Block 1.3 Digestive System 2009)

"These were common sentences we found during analyzing the learning objectives across all blocks in the year 1,2,3 undergraduate curriculum. Most of patient-centered care messages in the scenarios might be neglected when students or tutors found out these learning objectives. We understand that the basic medical sciences need these learning objectives. However, we think that most of learning objectives we analyzed were focusing only on area 2 of competency that was '*medical knowledge*' and abandon the other 6 areas of competencies of medical doctors in Indonesia that we understand from the Indonesian Medical Council, 2006".

(Agreement among three coders)

## Discussion

This study found gaps in expected patient-centered care values (presented in the scenario) and mostly disease-oriented actual learning objectives (presented in tutors' book). We have assumed that patient-centered care principles must have included in the 7 general areas of competences because the curriculum was oriented towards family medicine. The spirit of patient-centered care may have been missing along the way of the development of the curriculum and educational goals in the particular medical school. However, the way block planning groups creatively translated the disease-oriented learning objectives into more patient-centered scenarios were remarkable and included the effort of hospital specialists' who were trying to be aware of the importance of patient-centered care ideals. Clinical teachers created a more patient-centered scenario in purpose to attract students' attention to learn contextual cases. Three decades experience of problem based learning approaches of the medical school reflected in this endeavor of teaching patient-centered care values.

Nevertheless, there are threats to misuse the patient-centered care scenarios. First, the meaning of "contextual" clinical case. Teachers are mostly working at a teaching hospital. Lots of scenarios in later years are referring to a patient who came to the hospital. If the patient mentioned in the scenario presented in a primary health care settings, the disease description remains the same. It is not our goal to limit clinical knowledge but rather to put it into a context in which the patient is seen as more than the disease. Additionally, many presentations in primary care remain undifferentiated. It is important to teach clinical problem solving to students in order to better deal with this uncertainty [22]. In this respect, achievement of comprehensive and continuous care values is questioned.

Second, "contextual" scenarios are threatened by the current learning objectives. Students did not know the learning objectives when they read a scenario during a tutorial discussion. Therefore, most of tutorial discussions may begin with curiosity on "who the patient is" and "what was going on the context of clinical cases" as expected by the block planning groups. However, the curiosity on patients' background and concern may not last long. In the end of the discussion students will likely to formulate learning goals that are leaned towards diseases than illnesses as guided by tutors who have been guided by the learning objectives. Teachers in earlier year are mostly come from pre-clinical departments and are general practitioners. However, more than half of scenarios in the first year are leaned towards basic medical sciences of anatomy, physiology and biochemistry than to understand a family as a unit of care. Therefore, in three years of basic medical education, general primary care principles are likely insufficiently covered. Our observations on several actual learning outcomes of tutorial discussion formulated by students of academic year 3 showed limited attention to patient-centered care values. Most of actual learning outcome emphasized diagnosis and treatment establishment, without touching doctor - patient and their family - interaction. However, this observation was limited in number to be included in this study.

Another study in the area of effective communication showed that doctors in a teaching hospital rarely respond to patients' concern but mostly did brief diagnosis establishment and prompt treatment [23]. Further study on our community based program showed that students had difficulties in working with health workers and lay people in the area of interpersonal skills and inter-professional skills [24]. Thus, the current undergraduate orientation towards family medicine is ineffective.

We assumed that if we included year 4 tutorial discussions that will be no significance change to our findings. This raises the concern of unmet educational goal of patient-centered care in later years of study. Moreover, year 5 and 6 are clinical rotation stages which are department based (without a family medicine department). A medical doctor trained in this context of the study may have difficulty framing the problem of one of the patient's concern rather than a simple disease to be cured. Therefore, a clear distinction between studying basic medical sciences, studying general clinical medicine, and studying hospital-based clinical sciences is needed in the context of this study [25,26].

One of limitations in this study is that we analyzed a curriculum of only one medical school in Indonesia. However to our knowledge, this medical school leads many medical institutions in curriculum reformation. Another limitation was that only one method of discourse analysis performed. A more multifactorial methodology may leads to improved comprehension of the curriculum and prospectus modification [27]. The other limitation was that all GPs as coders in this study were not trained in a formal general practice specialization program, due to the fact that there is not such a program in Indonesia. The comprehension of the patient-centered principles could therefore be superficial. Studies in students' actual learning outcomes and students' assessment concerning patient-centered care values are essential following this study.

The current health problems in Indonesia are TB-HIV, high Maternal Mortality Rate and Infant Mortality Rate, Malaria, Dengue Fever, Malnutrition and Chronic illness such as Hypertension and Diabetes Mellitus [28]. Basic sciences should also view such cases from a general primary care medicine point of view which is different from hospital-based clinical perspectives. More community based educational approaches may help to sensitize medical students to be aware of different types of health problems in family, community and hospitals. Community based educational strategy had been proven to help students and teachers to understand the comprehensive care and continuous care principles [29]. By learning at the community settings, students are likely to perform direct observations of health care access, listening and responding to persons' concern, assisting people through various levels of preventions, seeing clinical cases from variation of clinical stages, working with people from different background and many more benefits help them sensitive with patient-centered care values.

## Conclusion

The current basic medical education curriculum in Indonesia that is supposed to be oriented towards family medicine has not succeeded. We recommended appropriate patient-centered care training for medical students to be more sensitive to patients' problem. Proper undergraduate and postgraduate training on primary care practice with community based educational approaches are required.

## Competing interests

Non-financial competing interest: The authors declare an academic competing interest of teaching family medicine with patient-centered care approaches for both undergraduate and postgraduate medical education.

## Authors' contributions

MC designed this study and wrote the first draft of this paper. MC and ADS were two of the three coders. MAG provided advices on the content of patient-centered care in family medicine. AS contributed advices on the content of curriculum-discrepancy and guided the process of scientific writing. All four authors discussed and revised the first draft until the final version to be submitted.

## Ethical approval

This study was part of an Evaluation Study of the Curriculum Development of Faculty of Medicine Gadjah Mada University under the NPT-Project, 2010. The ethical approval was obtained from the Committee Ethics UGM under the Evaluation Study of the Curriculum Development FM UGM 2010.
